# Supplementary material for: Empirical methods for controlling false positives and estimating confidence in ChIP-Seq peaks
Source: BMC Bioinformatics. 2008 Dec 5;9:523. doi: 10.1186/1471-2105-9-523 (PMC2628906; doi:10.1186/1471-2105-9-523)
Supplement: Additional File 1 — A variety of html documents from the USeq web site detailing the available applications, their best usage, output file type descriptions, command line menus, etc. [file 1471-2105-9-523-S1.zip › USeqUserGuides/JavaDoc.zip!/JavaDoc/util/apps/MakeBioTools.html]

MakeBioTools


|  |  |  |
| --- | --- | --- |
| **Overview** \| **Package** \| **Class** \| **Use** \| **Tree** \| **Deprecated** \| **Index** \| **Help** | |  |
| **PREV CLASS**   **NEXT CLASS** | **FRAMES**    **NO FRAMES**     **All Classes** |  |
| SUMMARY: NESTED \| FIELD \| CONSTR \| METHOD | DETAIL: FIELD \| CONSTR \| METHOD |  |


---


## util.apps Class MakeBioTools

```
java.lang.Object
  util.apps.MakeBioTools
```

---

``` public class MakeBioTools extends java.lang.Object ```

Utility app to pull classes into folders and extract menus to html.

---

| **Field Summary** | |
| --- | --- |
| `static java.lang.String` | `menuHeader` |


**Constructor Summary**

`MakeBioTools()`


| **Method Summary** | |
| --- | --- |
| `static void` | `main(java.lang.String[] args)` |
| `boolean` | `makeLibrary()`             Makes the code library jar from the directoriesForLibJar array. |
| `void` | `makeMenuHTMLDoc()`             Generates the cmdLnMenus.html doc. |
| `void` | `makeMockJars()`             Makes mock Jar files. |
| `void` | `makeSource()`             Makes the code library jar from the directoriesForLibJar array. |

**Methods inherited from class java.lang.Object**

`equals, getClass, hashCode, notify, notifyAll, toString, wait, wait, wait`

**Field Detail**

### menuHeader

```
public static final java.lang.String menuHeader
```

**See Also:**: Constant Field Values


**Constructor Detail**

### MakeBioTools

```
public MakeBioTools()
```


**Method Detail**

### makeMockJars

```
public void makeMockJars()
```

:   Makes mock Jar files.

---


### makeMenuHTMLDoc

```
public void makeMenuHTMLDoc()
```

:   Generates the cmdLnMenus.html doc.

---


### makeLibrary

```
public boolean makeLibrary()
```

:   Makes the code library jar from the directoriesForLibJar array.
    Saves as bioToolsCodeLibrary.jar

---


### makeSource

```
public void makeSource()
```

:   Makes the code library jar from the directoriesForLibJar array.
    Saves as bioToolsCodeLibrary.jar

---


### main

```
public static void main(java.lang.String[] args)
```


---


|  |  |  |
| --- | --- | --- |
| **Overview** \| **Package** \| **Class** \| **Use** \| **Tree** \| **Deprecated** \| **Index** \| **Help** | |  |
| **PREV CLASS**   **NEXT CLASS** | **FRAMES**    **NO FRAMES**     **All Classes** |  |
| SUMMARY: NESTED \| FIELD \| CONSTR \| METHOD | DETAIL: FIELD \| CONSTR \| METHOD |  |


---
